# Acute effect of healthy walking on arterial stiffness in patients with type 2 diabetes and differences by age and sex: a pre-post intervention study

**DOI:** 10.1186/s12872-019-1039-x

**Published:** 2019-03-08

**Authors:** Rosario Alonso-Domínguez, José I. Recio-Rodríguez, Maria C. Patino-Alonso, Natalia Sánchez-Aguadero, Luis García-Ortiz, Manuel A. Gómez-Marcos

**Affiliations:** 1 The Alamedilla Institute of Biomedical Research of Salamanca (IBSAL), Primary Health Care Research Unit, La Alamedilla Health Center, Salamanca, Spain; 2 Health Service of Castilla y León (SACyL), Salamanca, Spain; 3 Spanish Network for Preventive Activities and Health Promotion (redIAPP), Salamanca, Spain; 4 Faculty of Health Sciences, University of Burgos, Burgos, Spain; 5 Department of Statistics, University of Salamanca, Salamanca, Spain; 6 Department of Biomedical and diagnostic sciences, University of Salamanca, Salamanca, Spain; 7 Department of Medicine, University of Salamanca, Salamanca, Spain; 8 Salamanca, Spain

**Keywords:** Arterial stiffness, Type 2 diabetes mellitus, Walking

## Abstract

**Background:**

Daily aerobic exercise such as healthy walking could have an immediate effect on parameters of arterial stiffness; however, there is little evidence in the diabetic population. Our aim, therefore, is to evaluate the association between healthy walking and acute effects on the parameters of arterial stiffness in subjects with type 2 diabetes.

**Methods:**

The Effectiveness of a multifactorial intervention in diabetics study (EMID), is a study based on an application for smartphones, healthy walking and a nutritional workshop in patients with type 2 diabetes in primary care, is a randomized controlled trial of two parallel groups. This is a subanalysis of the intervention group to evaluate the response to the healthy walking according to age and sex, in 89 subjects with type 2 diabetes, aged between 40 and 70 years. The intervention was a 4 km of a healthy walking at low-moderate intensity. To value our aim, the main study variables were measured before and after it.

**Results:**

The study population had an average age of 65.0 years (61.2–68.1). After the healthy walking, there was a decrease in the parameters of arterial stiffness: Cardio ankle vascular index (CAVI) of − 0.2 (95%CI:-0.4 to − 0.1) and pulse pressure (PP) of the lower extremities of − 3.9 mmHg (95%CI: -5.9 to − 2.0). Furthermore, in the lower extremities there was a decrease in systolic blood pressure of − 5.3 mmHg (95% CI: -7.3 mmHg to − 3.3 mmHg), in diastolic blood pressure of − 1.5 mmHg (95% CI: -2.6 mmHg to − 0.4 mmHg) (*p* < 0.05 for all). It is observed that males have an OR of 2.981 (IC = 95% 1.095 to 8.119) to achieve a reduction in the CAVI (*p* < 0.05) and an OR of 2.433 (95%CI: 0.871 to 6.794) in the ankle PP (*p* > 0.05), compared with females.

**Conclusions:**

The findings of this study suggest that daily aerobic exercise at a low to moderate intensity, such as healthy walking, has an immediate beneficial effect on the cardio-ankle vascular index, especially in males.

**Trial registration:**

ClinicalTrials.gov identifier: NCT02991079.

## Background

Cardiovascular disease is the main determinant of morbidity and mortality in patients with diabetes [1]. The presence of diabetes accelerates vascular remodelling, leading to an increase in arterial stiffness and, as a result, the development of atherosclerotic diseases such as ischaemic cardiopathy, peripheral artery disease or carotid stenosis [2]. There is evidence that an increase in physical activity is associated with a decreased cardiovascular risk [3, 4] and has a beneficial effect on glycaemic control [5]. Liu et al. [6] concluded that measuring the immediate effect of exercise is a method to predict the magnitude of blood pressure-lowering after chronic training.

The protective effect of physical exercise on the cardiovascular system has been widely investigated, however, the immediate effect of the exercise on arterial stiffness is not known with certainty. In the study conducted by De Van et al. [7], resistance exercise produced a transient increase in arterial stiffness. However, Tabara [8], found a reduction in arterial stiffness after 30 min of aerobic exercise. According to Wang et al. [9], the intensity of the exercise is the most important variable, which determines the changes in stiffness. This author demonstrated that interval exercise at low intensity decreased the cardio ankle vascular index (CAVI), a parameter that assesses central and peripheral arterial stiffness independently of the blood pressure (BP) at the time of measurement [10].

The findings on the acute effect of the exercise and its magnitude on BP are contradictory, presumably due to the differences in sex and age in the different studies [11]. Nickel et al. [12], found a transient increase in the values of BP in older adults. Tibana et al. however [13], found a decrease in both systolic blood pressure (SBP) and diastolic blood pressure (DBP) in middle-aged females. Furthermore, the immediate reduction of BP after exercise has been studied in hypertensive and prehypertensive patients [14, 15], however, there is no evidence in the patients with type 2 diabetes.

Daily aerobic exercise such as healthy walking could have an immediate effect on parameters of arterial stiffness in the population with cardiovascular risk. However, there is less evidence of the acute effect of the exercise in subjects with diabetes, considering also subgroups of age and sex. Therefore, this study aims to evaluate the association between healthy walking and the acute effect on the parameters of arterial stiffness in patients with type 2 diabetes and to analyse the differences for age and sex.

## Methods

### Design and study setting

The Effectiveness of a multifactorial intervention in diabetics study (EMID) [16], is a study based on an application for smartphones, healthy walking and a nutritional workshop in patients with type 2 diabetes in primary care, is a randomized controlled trial of two parallel groups, with a follow-up period of 12 months. This is a subanalysis of the intervention group to evaluate the response to the healthy walking according to age and sex. The study was conducted in a primary healthcare setting at La Alamedilla research unit in Salamanca, which belongs to the Network for Research on Preventive Activities and Health Promotion (REDIAPP) and to the Biomedical Research Institute of Salamanca (IBSAL).

### Participants

This study analysed 89 patients with type 2 diabetes attending La Alamedilla healthcare center. The inclusion criteria were: patients with type 2 diabetes of both sexes, routinely monitored at the health care center, aged 40–70 years who, after receiving information about the study, agreed to take part and signed the informed consent. The inclusion criteria were: patients with type 2 diabetes of both sexes, routinely monitored in the health care center, from 40 to 70 years of age who, after receiving information about the study, agreed to participate and signed the informed consent. The exclusion criteria were: history of cardiovascular events (acute myocardial infarction, cerebrovascular accident, etc.); documented musculoskeletal, neurological and/or neuropsychological illness, which prevented carrying out the walk; or any other circumstance that, being properly evaluated by the researchers, could interfere with the proper development of the study.

With the 89 subjects included, and considering an alpha risk of 0.05 and a common standard deviation of the CAVI of 1.25, our study would have a contrast power for paired groups of 0.62, to detect a difference as statistically significant between the pre-intervention measure (8.6) and the post-intervention (8.3), of 0.3 units.

### Masking

Due to the nature of the intervention, the participants could not be blinded. However, the researcher who carried out the intervention in the study group was different from the person responsible for assessment and standardized counseling. The person responsible for carrying out the measurements before and after the intervention of all the participants was blinded to the objective of the study, to avoid bias. In addition, the person responsible for statistical analysis remained blinded throughout the study.

### Exercise intervention

The intervention involved a healthy walking of 4 km, on level ground, leaving from and returning to the health care center, accompanied at all times by health care nursing. The intervention was always conducted at the same time (5 p.m.). These measurements were carried out, having spent at least 2 h since their last meal. In order to make the walks qualify as aerobic exercise (50–70% maximum heart rate) [17], participants were divided into two groups depending on intensity. Approximate speed of the group walking at moderate intensity (5 METs) was 6 km/hour compared to 3–4 km/hour in the group walking at low intensity (2.5 METs) [18].

### Data collection

To evaluate the acute effects of the healthy walking, the main study variables were measured before and immediately after it.

#### Cardio ankle vascular index, brachial ankle pulse wave velocity and blood pressure

These parameters were estimated using the Vasera device VS-1500 (Fukuda Denshi) after entering of the participants’ information. These measurements were carried out, having spent at least 2 h since their last meal, with the patien in supine position after resting for 10 min in a quiet room at a stable temperature. CAVI integrates the cardiovascular elasticity derived from the aorta to the ankle pulse velocity through an oscillometric method. The CAVI values were automatically calculated by estimating the stiffness parameter β in the following equation: β = 2ρ × 1/(Ps–Pd) × ln (Ps/Pd) × PWV^2^, where ρ is the blood density, Ps and Pd are SBP and DBP in mmHg, respectively, and the PWV is measured between the aortic valve and the ankle (considered a measure of central and peripheral arterial stiffness) [19]. The average coefficient of the variation of the CAVI is less than 5%, which is small enough for clinical use and confirms that the CAVI has favourable reproducibility [20]. The ba-PWV was estimated using the following equation: ba-PWV = (0.5934 × Height (cm) + 14.4724)/tba, (tba is the time interval between the arm and ankle waves) (considered a measure of central and peripheral arterial stiffness) [21]. The CAVI was classified as normal (CAVI < 8), borderline (CAVI ≥8 or < 9), or abnormal (CAVI ≥9). Abnormal CAVI represents subclinical atherosclerosis [22–24]. In addition, with Vasera VS-1500 we obtained the measurements of the blood pressure (using the oscillometric method) and the heart rate (with an electrocardiogram meter).

#### Blood glucose measurements

A determination of capillary blood glucose was performed, with at least 2 h having elapsed since eating, using the glucose-meter *GlucoMen LX PLUS* (A. Menarini GmbH) [25].

### Other variables and measurement instruments

Before performing the intervention, the sociodemographic and lifestyle variables were collected: age, sex, marital status, educational level and toxic habits (tobacco and alcohol consumption). On the other hand, data were collected on the consumption of drugs, as well as family and personal history of risk factors: hypertension, defined as the use of antihypetensive drugs and/or systolic blood pressure ≥ 140 mmHg or diastolic blood pressure ≥ 90 mmHg, measured under basal conditions and making the average of three records; dyslipidemia, described by the consumption of lipid-lowering drugs, as well as, total cholesterol higher than 239 mg/dl or serum triglycerides higher than 199 mg/dl.

#### Anthropometric variables

These values were measured with the subjects barefoot and wearing light clothing. The weight was measured twice (the final result being the average of these) with a certified electronic balance (Scale 7830; Soehnle Professional GmbH & Co, Backnang, Germany), after having been adequately calibrated with an accuracy of ±0.1 kg. The height was measured twice (the final result was the average of these) with the subject standing, using a portable system (Seca 222; Medical scale and measurement systems, Birmingham, United Kingdom). Body mass index (BMI) was calculated by dividing the weight in kg by the square of the height (m^2^). We obtained the measurement of waist and hip circumference, following the latest recommendations of the SEEDO [26], and using a flexible measuring tape placed parallel to the floor.

#### Laboratory variables

Venous blood sampling were collected at the primary healthcare center between 08:00 and 09:00. To make these determinations properly, participants were informed that they should be fasting and should not have consumed tobacco, alcohol or caffeine during the previous 12 h. Subsequently, they were sent to the University Hospital of Salamanca to perform the analysis of creatinine, serum total cholesterol, low density lipoprotein cholesterol, high density lipoprotein cholesterol, triglyceride levels and glycosylated haemoglobin.

#### Physical activity

Regular physical activity was recorded using the short version validated in Spanish of the International Physical Activity Questionnaire (IPAQ) [27]. This questionnaire evaluates the physical activities of the last 7 days and classifies them into three types (walking, moderate and intense physical activity), according to their energy expenditure (3.3, 4.0 and 8.0 MET, respectively). This allows to calculate METs-min/week and classify people by three activity levels (low, intermediate and high).

### Data analysis

Statistical normality was checked using the Kolmogorov–Smirnov test. Normally distributed continuous variables were expressed as mean ± standard deviation, while non-normally distributed variables were presented as median and 25–75th percentile. T-Student or U Mann-Whitney tests were accordingly used to test the relationship between quantitative variables with Bonferroni correction. Categorical variables were presented as the frequency distribution and compared using the chi-square or Fisher’s exact test when necessary. Pre and post comparison analyses were carried out, using the Student’s t-test for paired data. A logistic regression analysis was performed considering the CAVI and the ankle pulse pressure as dependent variables (reduction = 1, no reduction = 0) and as independent variables sex (male = 1, female = 0), physical activity (METs/min/week), DBP (mmHg), BMI (kg/m^2^), age (years), hypertension (hypertensive subject = 1; non-hypertensive subject = 0), dyslipidemia (dyslipidemic subject = 1; non-dyslipidemic subject = 0) and baseline CAVI value or baseline ankle pulse pressure value (mmHg). For the two-sided tests, an alpha risk of 0.05 was set as the limit of statistical significance. The data were analysed using the IBM SPSS Statistics for Windows version 23.0 (Armonk, NY: IBM Corp).

## Results

Table 1 shows the characteristics (overall and by sex) of 89 patients with type 2 diabetes, with an average age of 65.0 years (61.2–68.1), of which 52.8% are hypertensive, 59.9% dyslipemic and 10.1% smokers. The mean BMI was 29.4 (29.2 ± 4.4 in males and 29.6 ± 4.1 in females). Physical activity in males is greater than in the females (1696.0 METs-min/week vs 1406.0 METs-min/week). 47.2% of the subjects performed the healthy walking at a low intensity, while 52.8% performed it at a moderate intensity. There are no significant differences between males and females in the studied variables. Furthermore, general anthropometric and clinical characteristics, by age (≤64 years or ≥ 65 years), are presented in Table 2.

**Table 1:** Baseline demographic and clinical characteristics of patient by genre

	Overall		Males (*N* = 52)		Females (*N* = 37)		*p*-value
	Mean/ Median/ Number	SD/IQR/(%)	Mean/ Median/ Number	SD/IQR/(%)	Mean/ Median/ Number	SD/IQR/(%)	
Age (years)	65.0	61.2–68.1	64.4	59.4–68.4	65.9	62.3–67.9	0.474
BMI (Kg/m^2^)	29.4	4.2	29.2	4.4	29.6	4.1	0.693
Office systolic blood pressure (mmHg)	132.6	16.4	130.9	17.0	135.0	15.5	0.241
Office diastolic blood pressure (mmHg)	80.5	9.0	79.5	9.0	82.0	9.0	0.200
Pulse pressure (mmHg)	51.5	41.3–59.3	49.8	40.5–56.3	53.0	42.5–63.3	0.589
Heart rate (beats/min)	72.3	11.8	72.0	12.1	72.8	11.6	0.736
Smoker n (%)	9	10.1	3	5.8	6	16.2	0.388
Physical activity (METS-min/week)	1605.0	777.5–2785.5	1696.0	930.8–2928.8	1406.0	516.0–2772.0	0.098
Intensity of the walk							0.816
- Low	42	47.2	24	46.2	18	48.6	
- High	47	52.8	28	53.8	19	51.4	
Hypertensive n (%)	47	52.8	30	57.7	17	45.9	0.274
Dyslipemic n (%)	53	59.6	32	61.5	21	56.8	0.205
HbA1c %	6.6	6.0–7.3	6.5	6.0–7.4	6.8	6.0–7.3	0.983
Serum glucose (mg/dl)	118.0	100.0–138.0	115.5	96.0–135.5	128.0	104.5–145.5	0.141
Total Cholesterol (mg/dl)	179.7	27.92	184.4	24.1	173.0	31.7	0.056
Triglycerides (mg/dl)	110.0	82.5–140.0	109.0	76.0–141.0	113.0	85.0–140.5	0.736
HDL Cholesterol (mg/dl)	55.0	14.2	55.3	14.5	54.5	13.9	0.801
LDL Cholesterol (mg/dl)	102.5	26.9	106.6	24.3	96.6	29.6	0.084
Antihypertensive drugs n (%)	46	51.7	28	53.8	18	48.6	0.234
Lipidlowering drugs n (%)	52	58.4	28	53.8	24	64.9	0.299
Antidiabetic drugs n(%)	81	91.0	46	88.5	35	94.6	0.319

Values are means [standard deviations (SD)] for normally distributed continuous data and medians (interquartile range) for asymmetrically distributed continuous data

*BMI* Body mass index, *METs* Metabolic equivalent, *HbA1c* hemoglobin A1c, *HDL* high-density lipoprotein, *LDL* low-density lipoprotein, *SD* Standard Deviation, *IQR* Interquartile Range

**Table 2:** Baseline demographic and clinical characteristics of patient by age

	≤64 years (*N* = 47)		≥ 65 years (*N* = 42)		*p*-value
	Mean/ Median/ Number	SD/IQR/(%)	Mean/ Median/ Number	SD/IQR/(%)	
Age	60.5	55.8–63.3	68.1	66.8–68.8	< 0.001
Sex	16	38.1	21	44.7	0.535
BMI (Kg/m^2^)	29.3	3.8	29.4	4.6	0.924
Office systolic blood pressure (mmHg)	131.6	16.5	133.5	16.4	0.575
Office diastolic blood pressure (mmHg)	81.0	9.2	80.0	9.0	0.608
Pulse pressure (mmHg)	52.0	40.9–56.3	51.0	41.5–64.5	0.326
Heart rate (beats/min)	73.1	11.2	71.6	12.4	0.567
Smoker n (%)	4	9.5	3	6.4	0.542
Physical activity (METS-min/week)	1689.3	779.8–2772.0	1406.0	757.0–2972.0	0.745
Intensity of the walk					0.940
- Low	20	47.6	22	46.8	
- High	22	52.4	25	53.2	
Hypertensive n (%)	23	54.8	24	51.1	0.731
Dyslipemic n (%)	25	59.5	28	59.6	0.996
HbA1c %	6.6	6.0–7.3	6.6	6.0–7.4	0.725
Serum glucose (mg/dl)	116.0	98.8–140.5	122.0	100.0–138.0	0.977
Total Cholesterol (mg/dl)	180.7	29.5	178.8	26.7	0.753
Triglycerides (mg/dl)	106.0	74.0–128.0	112.0	83.0–149.0	0.357
HDL Cholesterol (mg/dl)	53.9	12.2	56.0	15.8	0.505
LDL Cholesterol (mg/dl)	104.4	29.4	100.8	25.6	0.526
Antihypertensive drugs n (%)	18	42.9	28	59.6	0.118
Lipidlowering drugs n (%)	22	52.4	30	63.8	0.279
Antidiabetic drugs n(%)	37	88.1	44	93.6	0.369

Values are means [standard deviations (SD)] for normally distributed continuous data and medians (interquartile range) for asymmetrically distributed continuous data

*BMI* Body mass index, *METs* Metabolic equivalent, *HbA1c* hemoglobin A1c, *HDL* high-density lipoprotein, *LDL* low-density lipoprotein, *SD* Standard Deviation, *IQR* Interquartile Range

The differences in the parameters before and after performing the healthy walking by sex are shown in Table 3. There was a decrease in the CAVI values of − 0.2 (95% CI: -0.4 to − 0.1) (*p* = 0.012), although this only reached statistical significance in males − 0.3 (95% CI: -0.5 to − 0.1) (*p* = 0.012), but not in females − 0.1 (95% CI: -0.5 to 0.2) (*p* = 0.299). Furthermore, in the lower extremities there was a decrease in SBP of − 5.3 mmHg (95% CI: -7.3 mmHg to − 3.3 mmHg) (*p* < 0.01), in DBP of − 1.5 mmHg (95% CI: -2.6 mmHg to − 0.4 mmHg) (*p* = 0.007) and in the pulse pressure (PP) of − 3.9 mmHg (95% CI: -5.9 mmHg to − 2.0 mmHg) (*p* < 0.01). There was an increase in the HR of 9.2 bpm (95% CI: 7.2 bpm to 11.1 bpm) (*p* < 0.01) and a decrease in blood glucose of − 37.6 mg/dl (− 43.7 mg/dl to − 31.6 mg/dl) (*p* < 0.01). Comparing the changes of the different variables between males and females, none of these reached statistical significance.

**Table 3:** Effect of healthy walks on arterial stiffness, blood pressure and glycaemic, by sex

	Overall				Males (N = 52)				Females (N = 37)			
	Pre	Post	Difference (% CI)	*p*-value	Pre	Post	Difference (% CI)	*p*-value	Pre	Post	Difference (% CI)	*p*-value
CAVI	8.6 ± 1.2	8.3 ± 1.3	−0.2 (− 0.4; − 0.1)	0.012	8.6 ± 1.3	8.3 ± 1.2	− 0.3 (− 0.5; − 0.1)	0.012	8.5 ± 1.1	8.4 ± 1.4	− 0.1 (− 0.5; 0.2)	0.299
baPWV	15.7 ± 2.8	15.6 ± 2.6	−0.1 (− 0.5; 0.3)	0.580	15.7 ± 3.0	15.3 ± 2.4	− 0.4 (− 0.8; 0.1)	0.135	15.6 ± 2.7	15.9 ± 2.9	0.3 (− 0.3; − 0.8)	0.331
Heart rate	74.2 ± 12.6	83.4 ± 13.4	9.2 (7.2; 11.1)	< 0.001	72.4 ± 12.5	81.9 ± 13.6	9.5 (7.0; 12.0)	< 0.001	75.7 ± 11.6	85.4 ± 13.0	8.8 (5.5; 12.0)	< 0.001
Brachial SBP	136.4 ± 13.3	135.1 ± 13.5	−1.2 (−3.1; 0.7)	0.196	135.1 ± 14.0	133.9 ± 14.0	- 1.1 (− 3.8; 1.5)	0.384	138.2 ± 12.0	136.8 ± 12.7	− 1.4 (− 4.2; 1.5)	0.332
Brachial DBP	83.0 ± 8.4	82.8 ± 8.5	− 0.3 (− 1.5; 1.0)	0.660	81.6 ± 8.5	81.9 ± 7.9	0.3 (− 1.9; 1.6)	0.693	85.1 ± 7.9	84.0 ± 9.2	− 1.0 (− 3.3; 1.3)	0.366
Ankle SBP	155.8 ± 18.7	150.5 ± 17.6	−5.3 (− 7.3; − 3.3)	< 0.001	155.5 ± 18.5	150.3 ± 17.1	− 5.2 (− 7.8; − 2.6)	< 0.001	156.3 ± 19.2	150.9 ± 18.5	− 5.4 (−8.5; − 2.2)	< 0.001
Ankle DBP	77.2 ± 7.4	75.7 ± 8.2	−1.5 (− 2.6;-0.4)	0.007	76.4 ± 7.5	75.3 ± 8.5	−1.2 (− 2.6; 2.9)	0.116	78.3 ± 7.1	76.3 ± 8.0	− 2.0 (− 3.7; − 0.3)	0.021
Brachial pulse pressure	53.3 ± 9.7	52.4 ± 10.5	− 1.0 (− 2.7; 0.7)	0.259	53.5 ± 10.4	52.0 ± 11.3	− 1.4 (− 3.9; 1.0)	0.247	53.1 ± 8.8	52.8 ± 9.5	−0.4 (− 2.8; 2.1)	0.769
Ankle pulse pressure	78.6 ± 15.5	74.6 ± 15.0	−3.9 (− 5.9; − 2.0)	< 0.001	79.0 ± 15.1	74.7 ± 13.8	−4.4 (− 7.0; − 1.8)	< 0.001	77.2 ± 16.2	74.6 ± 16.7	−3.3 (− 6.3; − 0.4)	0.029
Glycaemic	159.1 ± 49.1	121.5 + 39.0	−37.6 (− 43.7; − 31.6)	< 0.010	157.9 ± 47.9	118.1 ± 40.5	− 39.8 (− 47.7; − 31.9)	< 0.001	160.7 ± 51.3	126.2 ± 37.6	− 34.5 (− 44.5; − 24.6)	< 0.001

*CAVI* cardio ankle vascular index, *ba-PWV* brachial-ankle pulse wave velocity, *SBP* systolic blood pressure, *DBP* diastolic blood pressure, *CI* confidence interval

Table 4 shows the differences in the parameters before and after performing the healthy walking by age. There was a decrease in the CAVI values, however, it only had statistical significance in the elderly − 0.3 (95%CI: -0.6 to − 0.0) (*p* = 0.033). On the other hand, as in the classification by sex, in the lower extremities, there was a decrease in SBP and in the PP in both groups, but only there was a decline in DBP of − 2.0 mmHg (95% CI: -3.3 mmHg to − 0.7 mmHg), in the older group. Moreover, there was a decrease in blood glucose in both groups, in the ≤64 years of − 37.9 mg/dl (95% CI: -47.6 mg/dl to − 28.2 mg/dl) and in the ≥65 years of − 37.3 mg/dl (95% CI: -45.3 mg/dl to − 29.4 mg/dl). Comparing the changes of the different variables between elders and minors of 65 years, none of these reached statistical significance.

**Table 4:** Effect of healthy walks on arterial stiffness, blood pressure and glycaemic, by age

	≤ 64 years (*N* = 47)				≥ 65 years (*N* = 42)			
	Pre	Post	Difference (95% CI)	*p*-value	Pre	Post	Difference (95% CI)	*p*-value
CAVI	8.5 ± 1.1	8.3 ± 1.0	− 0.2 (− 0.4; 0.1)	0.182	8.6 ± 1.3	8.3 ± 1.5	− 0.3 (− 0.6; − 0.0)	0.033
baPWV	15.7 ± 2.8	15.5 ± 2.4	−0.2 (− 0.7; 0.3)	0.495	15.7 ± 2.9	15.6 ± 2.9	−0.04 (− 0.6; 0.5)	0.881
Heart rate	73.1 ± 11.2	84.3 ± 12.6	8.4 (5.5; 11.3)	< 0.001	71.6 ± 12.4	82.5 ± 14.2	9.9 (7.2; 12.6)	< 0.001
Brachial SBP	134.9 ± 12.4	133.1 ± 11.8	- 1.7 (− 4.2; 0.7)	0.165	137.7 ± 14.0	137.0 ± 14.7	− 0.8 (− 3.7; 2.1)	0.583
Brachial DBP	84.0 ± 8.3	83.8 ± 8.2	− 0.2 (− 1.9; 1.6)	0.849	82.2 ± 8.5	81.8 ± 8.7	−0.4 (− 2.1; 1.4)	0.678
Ankle SBP	154.9 ± 16.1	150.7 ± 16.6	− 4.2 (− 6.9; − 1.5)	0.003	156.6 ± 20.9	150.3 ± 18.7	− 6.2 (− 9.2; − 3.3)	< 0.001
Ankle DBP	78.0 ± 7.0	77.0 ± 7.4	− 1.0 (− 2.7; 0.8)	0.287	76.6 ± 7.7	74.6 ± 8.9	−2.0 (− 3.3; − 0.7)	0.004
Brachial pulse pressure	50.8 ± 7.7	49.2 ± 7.9	−1.6 (−3.7; 0.5)	0.130	55.6 ± 10.9	55.1 ± 11.8	−0.4 (− 3.2; 2.3)	0.755
Ankle pulse pressure	77.0 ± 13.2	73.3 ± 14.6	−3.6 (− 6.3; − 1.0)	< 0.001	80.0 ± 17.2	75.8 ± 15.3	− 4.2 (− 7.0; − 1.4)	0.004
Glycaemic	152.8 ± 42.4	119.9 ± 30.1	− 37.9 (− 47.6; − 28.2)	< 0.001	164.7 ± 54.2	127.3 ± 45.5	− 37.3 (− 45.3; − 29.4)	< 0.001

*CAVI* cardio ankle vascular index, *ba-PWV* brachial-ankle pulse wave velocity, *SBP* systolic blood pressure, *DBP* diastolic blood pressure, *CI* confidence interval

In the logistic regression analysis (Table 5), it can be seen that being male has an OR of 2.981 (95% CI: 1.095 to 8.119) to obtain a reduction in CAVI (*p* < 0.05) and an OR of 2.433 (95% CI: 0.871 to 6.794) to obtain a decrease in the pulse pressure in the ankles (*p* > 0.05), after the healthy walking, compared to being female.

**Table 5:** Determinants in the improvement of arterial stiffness after a healthy walk

	Cardio ankle vascular index			Pulse pressure in the ankles		
	OR	95% CI	*p*-value	OR	95% CI	*p*-value
Sex	2.981	1.095 to 8.119	0.033	2.433	0.871 to 6.794	0.090
Age	0.978	0.897 to 1.066	0.614	0.989	0.907 to 1.079	0.809
Office diastolic blood pressure	1.039	0.982 to 1.100	0.182	1.035	0.975 to 1.099	0.263
Hypertension	0.531	0.183 to 1.543	0.245	0.769	0.276 to 2.269	0.663
Dyslipidemia	0.813	0.314 to 2.103	0.669	1.018	0.376 to 2.756	0.971
Physical activity	1.000	0.999 to 1.000	0.129	1.000	1.000 to 1.000	0.821
BMI	0.992	0.881 to 1.117	0.894	1.084	0.948 to 1.241	0.239
Baseline CAVI value	1.796	1.152 to 2.799	0.010	–	–	–
Baseline Pulse pressure value	–	–	–	1.037	0.999 to 1.075	0.055

Logistic regression analysis with CAVI decrease (0 = No decrease; 1 = decrease) and Pulse pressure decrease (0 = No decrease; 1 = decrease) as dependent variables and Sex (1: male; 0: female), age (years), office diastolic blood pressure (mmHg), Hypertension (1: Hypertensive subject; 0: Non-hypertensive subject), dyslipidemia (1: dyslipidemic subject; 0: non-dyslipidemic subject), physical activity (METs/min/week), BMI (kg/m^2^), baseline CAVI value or baseline ankle pulse pressure vale (mmHg), as independent variables

## Discussion

The main findings of the study were that the healthy walking of low-moderate intensity could have an immediate improvement of parameters of CAVI in patients with type 2 diabetes, especially in males and in people over than 65 years, since this is an inexpensive, simple and everyday exercise.

Various studies [9, 13, 27] support our results on the immediate beneficial effect that aerobic exercise could have on the parameters of arterial stiffness. Tabara et al. [8] found an association of the immediate effect of exercise with the long-term effects on parameters of arterial stiffness. Along these same lines, Madden et al. [28] found that aerobic training, on a treadmill and cycle ergometer, for 3 months produced an improvement in the arterial stiffness, despite not finding any improvement in other parameters such as weight, BP, BMI and waist-to-hip ratio. Yokoyama et al. [29] conducted a 3-week study, combining exercises on an ergometer and walking, which also found a decrease in arterial stiffness.

The intensity and type of exercise deserve key consideration when investigating the effect of exercise on arterial stiffness. Thus, in the study carried out by Mc Clean et al. [30], they found that no change had taken place in the parameters of arterial stiffness, since the intensity of the aerobic exercise had been insufficient. However, Wang et al. [9] found that low to moderate intensity aerobic exercise such as the healthy walking carried out in our study, produces a transient improvement in arterial stiffness, these results being supported by the meta-analysis performed by Ashor et al. [27].

In our study, the logistic regression shows that the only variable that could influence the change on the parameters of arterial stiffness after the healthy walking is sex; it being found that males are more than twice as likely as females to reduce the CAVI, compared to baseline, after the healthy walking. Some in vitro studies have described the action of estrogen on the vessels, in the reduction of smooth muscle proliferation [31] and in the increase in the release of nitrous oxide that leads to vasodilation [32]. Although the females studied are of an advanced age, and it can be assumed that they are in the post-menopausal state, there is evidence to suggest that differences due to sex in vascular biology are related not only to the type and levels of sex hormones, but also with the differences in the cells and in the tissues responsible for the responses to different stimuli [33]. Various studies have linked sex with differences in the CAVI, as occurs in our study, the CAVI being less in females than in males [34, 35], irrespective of their age.

Regarding the blood pressure, there was a decrease in both SBP and DBP in both lower extremities. These results are consistent with the meta-analysis conducted by Carpio et al. [11], where a reduction of 3 to 4 mmHg was found, confirming the importance of the immediate effect of exercise as a non-pharmacological method in reducing BP.

Considering the possible association, of the healthy walking used in our study, with the immediate beneficial effects on the CAVI, blood glucose and BP in the both lower extremities, this could be an activity to be recommended at primary care consultations, especially in males and in people over than 65 years, since this is an inexpensive, simple and everyday exercise. There was no improvement of BP in the upper extremities, this being because healthy walking exercise the both low extremities more intensely, furthermore, these are subjects with type 2 diabetes, a population more likely to have peripheral artery disease that causes a decrease in BP only in both lower extremities, after the exercise. We therefore believe that more studies are required to establish which exercises can be added to the healthy walking to produce cardiovascular improvement in both the upper and lower limbs.

This study has various limitations that need to be considered in the interpretation of our results. Firstly, the patients with diabetes included had multiple pathologies, and may be being treated with various drugs, which could modify the CAVI and BP values. We have tried to control this limitation through the inclusion of the most common drugs in the logistic regression. Secondly, being a pre-post intervention study, we had no control group with which to compare the data. Finally, the small size of the sample can make it difficult to find differences caused by the exercise.

## Conclusions

The findings of this study suggest that daily aerobic exercise at a low to moderate intensity, such as healthy walking, has an immediate beneficial effect on the Cardio-ankle vascular index, in patients with type 2 diabetes, especially in males.

## Abbreviations

ba-PWV: brachial-ankle pulse wave velocity
BMI: Body mass index
BP: Blood pressure
CAVI: Cardio ankle vascular index
CI: Confidence interval
DBP: Diastolic blood pressure
EMID: Effectiveness of a multifactorial intervention in diabetics
HDL: High density lipoprotein
HR: Heart rate
IBM: International Business Machines
IBSAL: Biomedical Research Institute of Salamanca
IPAQ: International physical activity questionnaire
LDL: Low density lipoprotein
MET: Metabolic equivalent
OR: Odds ratio
PP: Pulse pressure
redIAPP: Spanish Network for Preventive Activities and Health Promotion
SBP: Systolic blood pressure
SEEDO: Spanish Society for the Study of Obesity
SPSS: Statistical Package for the Social Sciences
